# Radiology AI training and assessment—challenges, innovations, and a path forward

**DOI:** 10.1093/bjro/tzaf026

**Published:** 2025-10-15

**Authors:** Girija Agarwal, Kavish Maroo, Paymon Zomorodian, Naman Bhatt, Dilan Sanli, Akash Sharma, Susan C Shelmerdine

**Affiliations:** Department of Clinical Radiology, St Mary's Hospital, Imperial College Healthcare Trust, London W2 1NY, United Kingdom; University College London Medical School, London, WC1E 6DE, United Kingdom; Department of Clinical Radiology, Mersey and West Lancashire Teaching Hospitals, Prescot, L35 5DR, United Kingdom; Department of Clinical Radiology, West Hertfordshire Teaching Hospital Trust, Hertfordshire, WD18 0HB, United Kingdom; Department of Clinical Radiology, University Hospital Southampton NHS Foundation Trust, Southampton, SO16 6YD, United Kingdom; Department of Clinical Radiology, St Mary's Hospital, Imperial College Healthcare Trust, London W2 1NY, United Kingdom; Department of Clinical Radiology, Great Ormond Street Hospital, London, WC1N 3BH, United Kingdom; UCL Great Ormond Street Institute of Child Health, Great Ormond Street Hospital for Children, London, WC1N 1EH, United Kingdom; NIHR Great Ormond Street Hospital Biomedical Research Centre, London, WC1N 1EH, United Kingdom

**Keywords:** radiology, artificial intelligence, ethics, education, assessment

## Abstract

Artificial intelligence (AI) is transforming radiology, with nearly 80% of approved AI as medical devices (AIaMDs) being imaging-related. As AI adoption accelerates, radiology training programs must evolve to equip future radiologists with the skills to critically evaluate, implement, and integrate AI into clinical practice. However, despite AI's growing role, its inclusion in medical curricula remains inconsistent, and assessment of AI competency is lacking. This review explores the current state of AI in UK medical training curricula with a more in-depth focus on radiology. We discuss the potential impact of AI on competency evaluations, including the Fellowship of the Royal College of Radiologists (FRCR) examinations, Annual Review of Competence Progression (ARCP), and on-call assessments. Additionally, we examine how AI-driven educational resources, such as AI-assisted training platforms, could enhance radiology education. To future-proof radiology training and careers, we propose strategies to evaluate AI literacy including nationalized structured AI teaching, and AI-focused assessments. Addressing these challenges will be crucial in ensuring that radiologists remain at the forefront of digital healthcare transformation while maintaining their core diagnostic expertise.

## Introduction

At present, the vast majority (almost 80%) of FDA and CE approved artificial intelligence (AI) as medical devices (AIaMDs) are related to radiology, with annual significant growth, and nearly 200 additional devices being added to the list in the last year alone.^1^ Nevertheless, without a clear understanding of how these digital innovations work, when to use them, and how to critically appraise their value, it will be challenging to adopt and trust these tools and translate their benefit to patient outcomes. The EU Artificial Intelligence Act underscores the importance of AI literacy, stating that providers and deployers of AI systems must ensure their staff achieve a sufficient understanding of AI.^2^ The UK government also acknowledges the importance of education and the need for AI ready healthcare staff having already funded several Topol Digital fellows (an NHS Digital Academy programme designed to equip fellows with new skills and knowledge to lead digital transformation),^3^ with Health Education England (HEE) part funding several Clinical AI Fellowships for trainees^4^ (focussed on AI implementation) and also publishing frameworks for how to improve staff confidence and trust in digital innovations.^5^ These directives support the argument that integrating AI into the curricula across all specialties is essential to prepare clinicians for the evolving demands of modern healthcare.^2^

Nevertheless, only a few medical specialties explicitly include AI in their curricula.^6^ In the United Kingdom, our resident doctors’ “Foundation programme”, and the specialist Radiology and Clinical oncology syllabi are some notable exceptions.^7–9^ They highlight that trainees should understand the principles of data analytics and AI—but currently do not assess these skills in any medical postgraduate examinations or appraisals. In contrast, other specialties such as cardiology and general practice do not explicitly require knowledge of AI but do encourage familiarity with “emerging technologies”.^10^^,^^11^ At present time, other specialty fields such as general surgery and internal medicine neglect mention of AI entirely from their training frameworks^12^^,^^13^ (Table 1).^6–17^

**Table 1.** Incorporation of AI/digital technologies into training curricula for different specialties.^6–17^

Specialty	AI included?	Notes/details
Clinical radiology	Yes	Includes AI and digital health in imaging and diagnostics
Clinical oncology	Yes	Includes AI and digital health in imaging and diagnostics
Foundation program	Yes	Includes understanding the principles of data analytics and AI
Cardiology	No	No explicit mention of AI; digital health tools are more broadly discussed
General practice	No	Lacks explicit AI; does touch on digital health tools
Psychiatry/general surgery/paediatrics/emergency medicine/internal medicine	No	No explicit reference to AI in the training curriculum
Dermatology	No	AI not included despite commercial autonomous AI diagnostic tools available

As AI continues to evolve rapidly, the role of radiologists (and other healthcare specialties) will inevitably change, with the expectation that they have the skills and awareness to effectively integrate and use AI safely and responsibly in their daily practice. Given the domination of commercial AI offerings in the radiology space, how we evaluate and train future radiologists for the demands of a digitally enhanced future as consultants, will be critical not only for them to train the next generation of doctors but also in leading the way for other medical specialties.

This thought leadership piece explores the potential challenges and solutions for evaluating AI knowledge across current radiology trainees, by radiology trainees and fellows with a keen interest in AI. Although this article focuses on UK radiology training, AI in education is a global concern and the discussions are generalizable to other countries as well.

## Current role of AI in medical education

Following medical school and a 2-year foundation program as a resident doctor (sometimes optional further years can also be taken), medical subspecialty training in the United Kingdom ranges from another additional 3 years for general practice or to up to another 8 years for other specialties, such as paediatrics.^18^

Radiology subspecialty training typically spans 5 years if undertaken full-time, during which trainees must meet curriculum requirements, pass Annual Review of Competence Progression (ARCP) assessments each year, and succeed in clinical radiology fellowship examinations to obtain a Certificate of Completion of Training (CCT). This curriculum ensures radiologists are competent in general and emergency radiology, in up to 2 subspecialty areas, and adept at tackling multidisciplinary team working, leadership, education, and management skills.^8^

In 2019, the Topol Review was published and emphasized the need for an increase in overall digital literacy within the NHS and of the general public to allow for an imminent digital health era. A key emphasis was made on the need to formally educate the medical workforce on AI, and develop relevant technology-related educational resources.^19^ In response, Health Education England (HEE) introduced “Knowledge for Healthcare”, an initiative to ensure that all NHS staff and learners have equitable access to high-quality knowledge services.^20^ Similarly, the 2021 clinical radiology curriculum was updated to include the expectation that trainees be able to critically appraise new technologies, including AI and machine learning applications.^8^ This encompasses understanding the data curation process, privacy and anonymization considerations, the limitations of AI based on its training data, and the basic concepts of radiomics, where radiomics is a quantitative analysis of radiology imaging.^21^

However, assessing these competencies remains challenging. Many trainees face increasing burdens to stay on top of their clinical knowledge, and it is easy to neglect this facet of the curriculum. Despite this, there is a great willingness and intention to learn, as highlighted in a Canadian study where 73% of oncology residents wished to learn more about AI^22^ and another study where 98.7% of UK radiology trainees felt that AI should be taught during their training.^23^ The issue with providing training and assessment however also comes from supervisors, who are still themselves learning about AI, and possibly feel unable to teach AI or judge a trainee’s competency.

Although the Royal College of Radiologists (RCR) is adapting to this new era by introducing AI-focused training courses such as the “Clinical Radiology Artificial Intelligence: A Blended Learning Program”,^24^ structured and mandatory education on AI is still lacking, and those who subscribe to the course must do so and pay for this out of their own pocket.

## Radiology specialty training assessments

The current methods of assessment of radiology trainees within the United Kingdom are outlined below with ideas on how AI may be evaluated through these or create issues for usual radiology competency evaluation in the near future.

### Annual review of competence progression

Plain film reporting is still a significant part of the radiology curriculum and trainees are expected to become proficient in reporting them. Although the exact number varies, many radiology training schemes expect trainees to report a certain number of plain films per year as part of the ARCP requirements. With the advent of AI for chest and musculoskeletal radiograph reporting, several challenges arise:

1. Firstly, if AI triages abnormal radiographs for urgent radiology review by either the consultant or reporting radiographer (and potentially report high confidence normal radiographs autonomously in the future), will there be enough examinations left for trainees to learn pattern recognition skills on these routine images? Furthermore, if enough radiographs remain, will trainees be disproportionately exposed to particular types of cases, potentially biasing their idea of normal and abnormal cases.
2. Secondly, if AI reports still require human sign-off, how should we implement it to still allow effective training of residents? Should trainees have access to AI output reports during their training, even though this might introduce bias? Or should a certain number of “no-AI” plain films be reserved exclusively for trainee teaching, free from AI assistance? The latter approach introduces the ethical issue of having plain films that are only human-reported, and would need to ensure that patient care is not being compromised. Another option may be to allow only fully independent reporters to be able to see the AI output, so residents would have to report independently of the AI. However, this would need to be feasible on a technical level, and would require intent and planning.

These questions will need to be addressed at both local and national levels to establish a standardized approach. At present, radiologists do still need to be proficient in reporting plain films, as current UK regulations require trained human readers to verify AI-generated reports.^25^ However, as the legal framework evolves, and AI becomes more reliable, radiographic reporting may become a less critical component of ARCP requirements or of the radiologist’s typical task or job role.

### Using AI to train radiologists

To address these challenges, we could potentially use the AI to teach us how to report rather than to take away opportunities.^26^ This would be particularly relevant in the age of workforce shortages, where healthcare professionals may lack appropriate time for teaching and training.^27^

**Figure 1.**
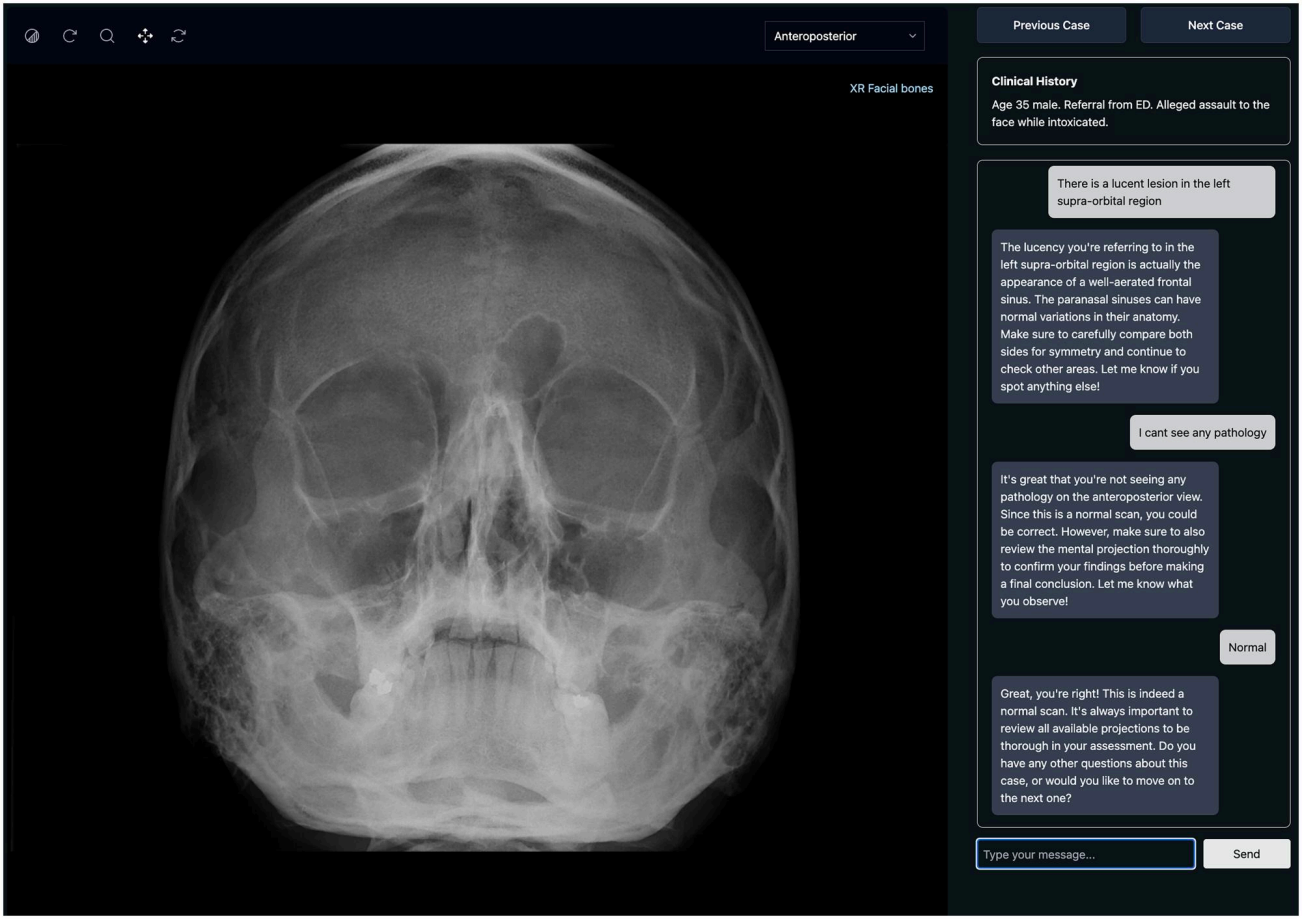
Screenshot of the RadBytes radiology AI tutor. The AI responds to the user’s comments about the image in a manner similar to a human tutor.

An example of an AI-driven radiology education platform is RadBytes, created by a radiologist whilst in training, which integrates AI to enhance radiology training and assessment. RadBytes has pioneered a Radiology AI Tutor, tailored to plain film interpretation. This interactive tutor delivers real-time, personalized feedback on trainee reports, meeting the needs of the individual. By simulating the guidance of a mentor, the system enables trainees to refine their diagnostic accuracy in a clinically simulated, self-directed learning environment (Figure 1).

**Figure 2.**
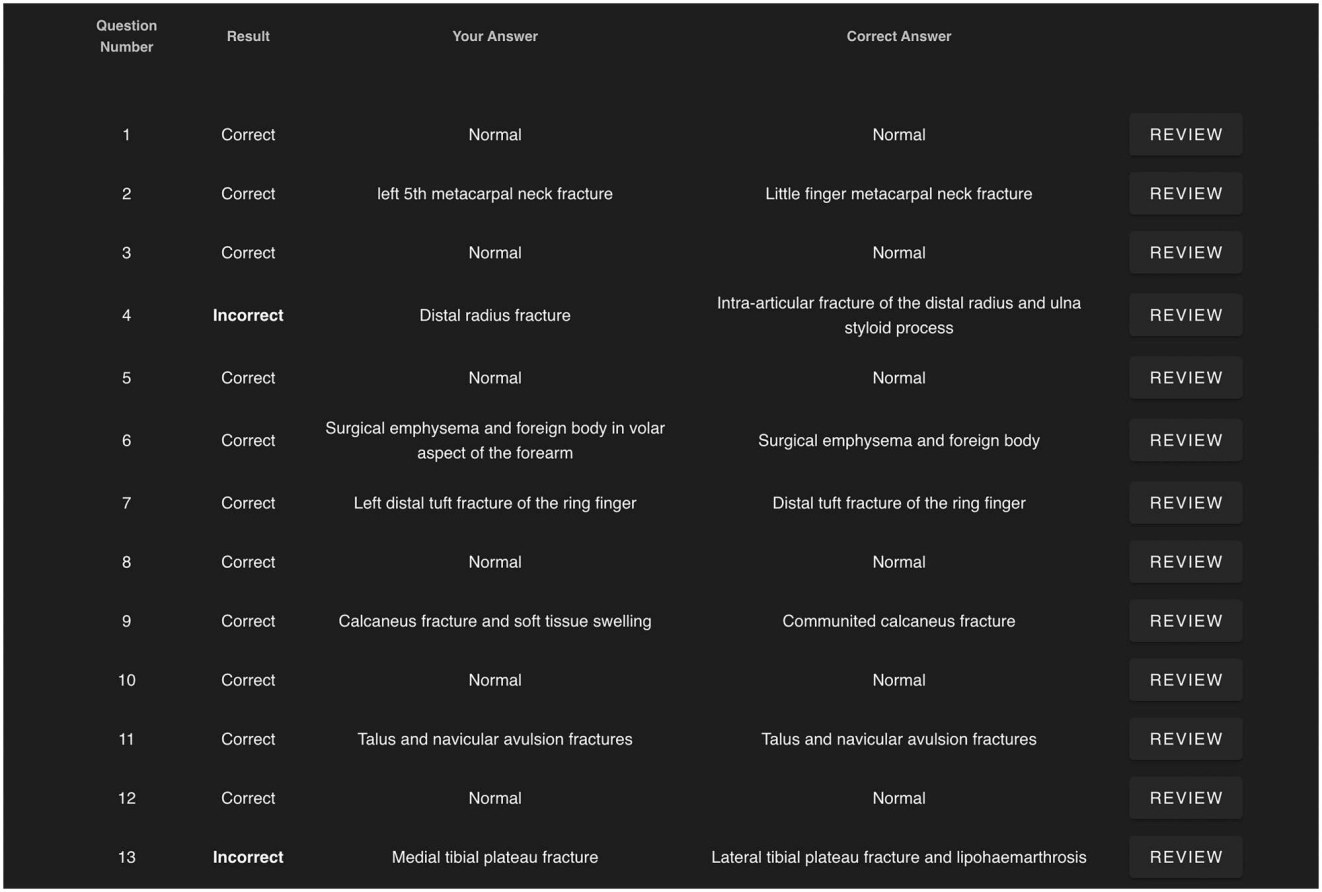
Screenshot of the RadBytes radiology AI tutor automatically evaluating the user’s response as correct or incorrect. The AI interprets nuanced answers, allowing for correct assessments even when the wording does not exactly match the expected response.

Another application developed is the use of AI to mark radiology examinations, streamlining the assessment process while improving accuracy and consistency. Marking imaging reports can be time-consuming and subject to inter-rater variability. By leveraging AI-driven scoring algorithms, RadBytes created a framework for a more objective evaluation while increasing self-marking efficiency (Figure 2).

While AI tutors such as RadBytes offer scalable, consistent feedback for trainees, they are not without limitations. RadBytes does not interpret images directly; instead, it uses a large language model (LLM), named Cubey, to analyse trainee-submitted reports or questions and provides feedback based on expert verified interpretations and a structured repository of radiological knowledge. Clinical history and supplementary information are provided to both the trainee and the AI, supporting context-aware feedback. However, the AI cannot directly point to or highlight pathology on the image itself, limiting its ability to visually guide trainees. Its feedback is also less adaptable than that of a human mentor, as it cannot interpret tone, assess confidence, or tailor its teaching to a trainee’s learning style or level of experience. Furthermore, disparities in access to such tools across deaneries could lead to inconsistent development of AI literacy among trainees. Such tools are best positioned to complement rather than replace critical thinking, supervision, and clinical judgement.

### “On-call” work

At a local level, some schemes require trainees to pass an on-call assessment before being allowed to provisionally authorize reports out of hours, ensuring they can identify urgent pathologies. However, with AI algorithms now capable of detecting stroke and intracranial haemorrhage, trainees may not encounter sufficient case load without AI influence, potentially affecting their independent learning. This is particularly relevant as radiologists who use AI must also be aware of the potential limitations of the software they use. In a UK study evaluating the accuracy of AI software in detecting abnormalities on CT angiograms for stroke patients, an error rate of 9% was found. This could clearly have significant impacts on patients.^28^

Again, in these situations—should trainees be prohibited from using AI until they prove their competence, or should training evolve to reflect the integrated AI workflows of the future?

Alternatively, another way of looking at this issue would be that the assistance of AI as a double reporter, may enable trainees to take on on-call duties earlier in their training to alleviate the out of hours burden on other more senior staff in the department and ease rota issues. This may also introduce them to the demands of prioritizing urgent cases and dealing with referrals sooner in their career to build their communication and interdisciplinary training skills. The AI would therefore play a powerful role in early education and confidence building.

When looking at translatable sectors where succeeding in a short space of time is crucial, similar models of work have been adopted. One such concept is that of “Lean Methodology” where commercial ventures follow an iterative process of failing fast, learning fast, and subsequently succeeding fast. In healthcare, the opportunity cost has often been deemed too high. However, with the advent of AI imaging reads, this may be something we adapt to out of necessity once the risks and mitigation strategies deem this route to be sufficiently robust.

### Fellowship examinations

Finally, the most comprehensive assessments in radiology training are the FRCR exams, comprised of three components (part 1, part 2A, and part 2B).

#### FRCR part 1

Part 1 test is an online multiple choice and image viewing exam undertaken at ST1 level (first year) of training consisting of anatomy and physics modules, and evaluates understanding of basic principles of medical physics and anatomy. Currently, this does not evaluate the understanding of AI or informatics but could be adapted in the future to cover this part of the curriculum to encourage trainees to understand AI basic principles from the very start of their radiology careers.^29^

#### FRCR part 2A

The second part, named 2A, is an online single best answer exam involving 240 multiple choice questions—this is predominantly theory based on medical diseases and imaging findings and clinical management and next steps across all radiology subspecialty fields.^30^

#### FRCR part 2B

The final part of the FRCR (named 2B) consists of 3 components. Firstly, an oral viva component where candidates are assessed on their interpretation of 12 cases. Secondly, a long cases examination where candidates write reports for 6 cases and, as of July 2025, a short cases examination component. The final short cases component has replaced the previous rapid reporting module and consists of 25 plain radiographs that candidates are expected to write reports for as well as recommend further management.

In comparison, the previous rapid reporting component involved identifying normal and abnormal radiographs with only brief descriptions of the abnormality. This change was made to more accurately reflect current radiological practice, as the candidates will now be provided with relevant clinical information, and it will also allow for more complex radiographs to be included. Although there is no mention of AI as a driving force for the change in the rapid reporting component, the new short cases will now require candidates to recommend further management plans and MDT referrals, which is in line with what would be the role of an AI-assisted radiologist.^31^ However, the question still remains—if training is in an environment where AI is used on a daily basis, should the AI be available in the exam itself?

#### Sub-specialist training

Most UK radiologists will be trained in a specific subspecialty, such as musculoskeletal or neuroradiology. Each subspecialty will bring its own hurdles to AI integration in training as some are more disrupted by AI than others. Paediatric radiology, for example, poses a unique challenge for AI training given the relative lack of regulated and available AI tools—due to the heterogeneity of the population, lack of large amounts of curated training data, and hence, may benefit from tailored human focussed education approaches. Breast radiology, particularly breast screening, may potentially be more impacted by AI given multiple large, multi-centre trials that may mean double reporting for screening mammograms could be a thing of the past.^32^^,^^33^ This could either mean fewer breast radiologists will need to be trained for reporting screening studies, but also that those who do wish to undertake this type of work will need to be more vigilant about potential AI pitfalls and when to override these.

#### Challenges with FRCR for AI literacy assessment

The current FRCR curriculum is designed around structured exam preparation and local teaching programmes, ensuring a standardized approach to radiology training. However, this structured framework leaves limited room for self-directed learning or exploration beyond the syllabus, particularly in emerging areas such as AI, which is increasingly shaping modern radiology workflows.

The Topol Review^19^ has highlighted the urgent need to upskill the radiology workforce to effectively engage with AI-enabled imaging infrastructure. To bridge this gap, NHS organizations should embrace a multi-professional learning approach, equipping radiologists with digital competencies necessary for AI integration into clinical practice. This could be achieved through a combination of formative and summative assessments, including workplace-based evaluations during clinical placements.

Moreover, revisiting AI concepts at multiple stages of training, using techniques such as spaced repetition,^34^ could reinforce understanding and retention. This iterative approach would ensure that radiologists are not only exam-ready but also prepared for the evolving demands of AI-assisted clinical practice. These proposed strategies for AI integration into radiology training are summarized in Table 2.^8^^,^^29–31^

**Table 2.** Proposed strategies for integrating AI education into radiology training in the UK.^8^^,^^29–31^

Assessment	Discussion
FRCR part 1	Comprised of anatomy and physics questions. Given the theoretical nature of the part 1 exam, core principles of AI could be included to ensure a basic level of common language is understood by trainees This could be as simple as a word-definition matching exercise, so trainees are familiar with common AI terminology and principles
FRCR part 2A	Single best answer questions that examine all aspects of clinical radiology, physics, anatomy, and techniques. In the future, this may change to include questions on critically appraising AI
FRCR 2B	Comprised of an oral viva station, a long-cases reporting station and, from June 2025, a short-cases reporting station which has replaced the previous rapid reporting module Although the short cases module is a new addition, it is likely to change the most, as this is entirely based on radiograph reporting and in the future, could include AI-assisted radiograph reporting with some cases where the AI is incorrect The reporting and viva stations could also give candidates access to the AI interpretation, so they must not only interpret the image but have knowledge of the pitfalls of AI and confidence to override the results where needed
On-call assessment	Locally organized examination to assess if trainees can spot common on-call pathologies. At this stage, trainees will be junior so it may be reasonable to ensure they can interpret imaging without AI assistance to build that capability
Independent reporting assessment	Local assessment for post FRCR trainees to decide if they can authorize reports without consultant checking. Potential integration of AI into assessments may be considered
Yearly ARCP assessments	Formal end-of-year portfolio review to see if trainees have met the curriculum requirements. This may change to have a section to show evidence of meeting AI-related curriculum requirements. For example, there may be mandatory AI training or an AI-audit introduced Additionally, if not already formally introduced into the FRCR exams, then basic principles of AI could be incorporated into ARCP requirements by an e-learning for health module Furthermore, hands-on experience with using AI tools in the clinical setting could be assessed using the existing portfolio (Mini-IPX and DOPS forms)
Final ARCP assessment	The final portfolio assessment, which leads to the Certificate of Completion of Training (CCT), could incorporate evidence of AI education and training, such as completion of an AI-related audit or research project. This would ensure that trainees have gained practical exposure to AI and its role in radiology To support the final decision on whether to award the CCT, an AI-driven Clinical Radiology Curriculum system could be developed to assess trainee readiness for consultancy. By automatically reviewing portfolio submissions, it could evaluate competency in key areas, track progress over time, and provide objective assessments to assist ARCP panels This system could also highlight strengths, identify areas needing improvement, and generate personalized training recommendations to ensure trainees meet curriculum standards before being awarded CCT By introducing AI-assisted evaluation, the process would become more consistent, data-driven, and tailored to individual training needs, ultimately enhancing both trainee development and the reliability of ARCP decisions

## AI teaching and training

Before introducing AI-related assessments into training, it is essential to ensure that trainees have access to sufficient learning resources. The RCR should take the lead in setting standards for education, and along with individual radiology academies and deaneries, provide teaching, guidance, resources, and mentorship to support AI education. This is particularly important given the limited number of AI-experienced radiologists and the uneven access to AI tools across different training regions.

Furthermore, the importance of interdisciplinary collaboration should be emphasized, including involving data scientists, informatics experts, or engineers in curriculum development and identifying joint training opportunities. As well as this, the RCR could collaborate with other institutions like European Society of Radiology (ESR) and Radiological Society of North America (RSNA) to bolster and inform the curriculum, as they too have been developing AI courses. Suggested resources are outlined in Table 3.^35^^,^^36^

**Table 3.** Suggested resources to facilitate AI literacy for doctors, and specifically radiologists.^35^^,^^36^

Resource	Discussion	Summary
Teaching	Structured teaching for trainees varies by region, such as the London deanery offering a monthly pan-London teaching schedule. In addition to this, regular formalized lectures on data science, AI basics, and evaluating AI tools could be incorporated into training. This method has potential as it seems to be the favoured approach of oncology residents in Canada to learning more about AI^35^ Trainees should also have access to AI teaching modules through the RCR or other educational platforms, such as courses offered by ESR and RSNA. These modules could cover key topics such as the stages of building an AI tool, FDA approval processes, EU regulations, legal responsibilities, and basic Python programming to enhance digital literacy Upon completing the AI curriculum, trainees could receive certificates or CPD credits, which could be included in their ARCP assessments. This would formalize AI education and ensure that trainees develop the necessary skills to integrate AI into their practice effectively	Structured teaching and formalized lectures AI modules Certificates and CPD for ARCP
Teaching day	The RCR could organize a “travelling professor in AI” training day for each deanery. The basics of AI, machine learning/deep learning can thus be delivered in one day by experienced AI enabled radiologists	Dedicated teaching days Travelling professor lectures
Training days	RCR-HEE-Industry away days could be organized several times a year, where trainees sign up to visit industry offices for knowledge exchange. These events would provide valuable networking opportunities and help trainees gain insights into the practical applications of AI and other technologies in healthcare. Similar to the NHS Clinical Entrepreneur Program, these away days would foster collaboration between trainees and industry experts Some opportunities like this already exist (eg, BiteLabs^36^), although this is outside the usual subspecialty training programmes	Away days with industry partners
Simulation	Training can also be delivered via simulated scenarios. There are simulation fellows in many deaneries and an AI simulation day can be arranged simultaneously between the Simulation Fellows, AI model builders (data scientists), AI fellows and trainees. The AI tutor created by RadBytes would be one such example of training simulation	AI simulation days with the multidisciplinary team
Conferences	In February 2025, the RCR hosted the first Global AI conference in collaboration with NHS England. Trainees could be further encouraged to present their projects, talk about their personal experience in AI as well as receive further AI training and simulation. There could be more streams or more opportunities to share implementation stories nationally here delivered by those on the frontline, organized by trainees, which would develop their leadership and management skills at the same time. This would also offer opportunities for them to network with other emerging AI leaders to call upon for support for future implementation challenges	Organizing and encouraging attendance at AI conferences
Mentoring	Having an AI mentor or supervisor throughout part or all of a trainee’s training can provide essential support from an expert This mentor could be a data scientist developing AI tools or an AI-enabled radiologist. With this guidance, trainees could engage in AI-related research, audits, and quality improvement projects, collaborate with AI experts, and participate in workshops and seminars to enhance their learning and practical experience This structured mentorship would help bridge the current knowledge gap and foster AI competency in radiology	AI mentorship to encourage research and quality improvement
Discrepancy meetings	A national AI-based REALM (Radiology Events and Learning Meetings) would allow trainees to discuss the strengths and weaknesses of AI in imaging assessments, learning from real-world examples nationally. These sessions would help them understand how AI works in practice, improving their ability to apply it in clinical settings	AI-based REALM meetings

## Training AI-literate consultants

As AI becomes more embedded in radiology, consultants must adapt by integrating validated AI tools into practice. This requires new competencies beyond traditional imaging skills, including AI fundamentals, data curation, algorithm training, radiomics, and statistical literacy to evaluate AI-driven research. Consultants must also understand AI biases, limitations, and data privacy implications.

Beyond diagnostic AI, emerging workflow tools are streamlining vetting, reporting efficiency, and MDT follow-ups. With the rapid evolution of AI, a structured educational approach is essential. One solution is establishing AI fellowships, training radiologists as “AI champions” who guide peers in clinical AI applications. As AI reshapes hospital workflows, a dedicated AI multidisciplinary team may emerge, as outlined in the 2021 NHS AI Lab and Health Education England (HEE) report.^5^

At a national level, the RCR is developing AI resources, such as an AI registry, and working with policymakers to shape its integration into radiology.^37^ However, is it realistic for the RCR to spearhead AI integration alone, given competing priorities like workforce planning and education? This raises the possibility of creating a united space for AI advisory boards of all Royal Colleges to gather (potentially enabled by the Academy of Medical Sciences) and share developments and ideas across their subspecialty areas.

Additionally, it is important to consider the training needs of mid- to late-career consultants, which may differ from those of early-career consultants entering the workforce. Having been trained without AI, they may be more hesitant to adopt such technologies. However, failing to upskill this group risks creating a divide within the profession—between AI-assisted radiologists and those relying solely on traditional, human-only methods.

## Management

Radiologists routinely engage in clinical governance, audit, and quality improvement (QI), fulfilling ARCP requirements. AI and LLMs can enhance these processes by analysing vast datasets for audits,^8^ but their use presents transparency, bias, and data confidentiality challenges. AI systems often function as “black boxes”, raising concerns about fairness and accuracy in decision-making.^38^

To ensure safe and ethical AI use, governance frameworks are emerging.^39^ For radiology trainees, understanding these frameworks is becoming as essential as traditional QI training. Incorporating AI-related audits and projects into the curriculum would familiarize trainees with AI governance, while consultant revalidation could require AI competency assessments, mirroring existing QI expectations.

## AI in pre-specialist training

As AI awareness grows, future radiologists will enter specialty training with greater AI exposure. Medical students today view AI positively and are not widely concerned about job displacement—a study of 293 individuals (211 medical students) found only 15% believed AI would significantly impact the physician job market.^40^ However, their AI knowledge and exposure remain inconsistent, as there is no national undergraduate AI curriculum.

Some medical schools have introduced AI education, often within ethics modules or self-directed digital health projects. Topics may focus on the challenges of data usage, privacy, and bias when training an AI model and the ethical implications of digital health equity. These examples are rare, however, with 91.4% of students in a study of 325 stating they had not received any training on AI in their medical curriculum.^41^

While most students have limited real-world AI exposure, many have used LLMs (eg, ChatGPT) for academic work. A small subset may have learned AI fundamentals through A-level computer science or physics, but they remain a minority.

To bridge this gap, integrating basic AI principles into medical education would help future doctors develop a realistic understanding of AI’s capabilities and limitations. However, this should not come at the expense of core clinical skills, ensuring that doctors rely on sound clinical judgement rather than overdependence on AI.

## Summary

We can summarize the unanswered questions briefly as below:

The evolving future role of a radiologist:

- What will the future role of a radiologist look like?
- How do we build inherent confidence and self-trust to know when to override erroneous AI results in practice and how do we reassure radiologists that they will not be (more severely) punished for errors when working with AI?
- What ongoing training and evaluation should be introduced for radiologists (both in training and after training) to account for automation bias and potential deskilling?

Redesigning training pathways:

- Should radiology residents in training have access to AI tools from the beginning of their training?
- Is there a role for AI in medical education and training? And to what depth?
- Should fellowship examinations and assessments be changed to account for the presence of AI tools in routine clinical practice?
- Speaking more broadly, should AI training (and even more basic computational sciences like healthcare informatics) be mandated/introduced into the medical undergraduate and pre-specialist training curricula?

Governance, equity, and standards in training:

- How can we upskill clinicians to become AI-literate, and what is our definition of AI literacy?
- Who should take responsibility and have oversight over training radiologists about AI? This is particularly pertinent where there may be a senior—junior divide in understanding of the digital landscape.
- How do we ensure a standardized and consistent level of AI training and experience and skills nationally when the integration and deployment of AI tools is not yet widespread across the NHS?

## Conclusion

AI is already having a significant impact in radiology, meaning training programmes and their directors must adapt to prepare future radiologists for this shift. Trainees need to learn how to integrate AI tools into their practice while maintaining core radiology skills. They need to be able to identify abnormalities without AI, be able to critically appraise the tools, and have the confidence to challenge the AI when they feel it is wrong. This would require updates to education resources, assessments, and possibly the introduction of AI-focused fellowships or specialized roles. This thought leadership piece has suggested some areas of challenge but also many opportunities on how AI could be assessed and integrated in augmenting radiologists of the future.

## Glossary of AI-related terms

Artificial intelligence (AI): A field of computer science focused on creating systems capable of tasks that normally require human intelligence, such as image interpretation or decision-making. This can be a broad terminology that covers many different actions and aspects, and therefore specific “subtypes of AI” may sometimes be quoted to be more specific and aid understanding (eg, LLMs, as below).

AI as a medical device (AIaMD): This terminology refers to AI systems that are regulated and approved for clinical use. These must meet safety and performance criteria by regulators and require conformity assessment and certification, such as CE marking (EU) or FDA clearance (USA). Tools that are not regulated for clinical use, should strictly not be deployed for this purpose.

Radiomics: A process of extracting quantitative data from medical images, such as shape or texture features, to support AI model training or clinical decisions.

Automation bias: The tendency to over-rely on AI system outputs, even when they may be incorrect, potentially undermining critical clinical judgement.

AI literacy: The knowledge and skills required to understand, evaluate, and appropriately use AI in clinical practice, including awareness of its limitations and risks.

Large language model (LLM): A type of AI model trained on large text datasets to generate human-like language. Examples include ChatGPT, used increasingly in research writing and communication.

Black box AI: A term used to describe AI systems where the internal logic or reasoning behind outputs is not transparent or easily understood, raising concerns around accountability and explainability particularly when erroneous or unexpected results are produced.

AI registry: A database tracking AI tools used in clinical settings, and potentially capturing their performance data. The purpose of this is to allow other users (or potential users) to know who to reach out to in order to get impartial and unbiased advice from (outside of the vendor), and potentially pool outcome data and comparisons across different populations.
